# The influence of dietary protein concentration on digestive enzyme activities, growth, and body composition in juvenile bullseye snakehead (*Channa marulius*)

**DOI:** 10.1371/journal.pone.0281274

**Published:** 2023-02-14

**Authors:** Sadia Nazir, Noor Khan, Mahroze Fatima, Hamda Azmat, Saima Naveed, Malik Muhammad Ramzan, Muhammad Asghar, Sheeza Bano, Ayesha Khizer, Alex H. L. Wan, Simon John Davies

**Affiliations:** 1 Department of Fisheries & Aquaculture, University of Veterinary and Animal Sciences, Lahore, Pakistan; 2 Department of Animal Nutrition, University of Veterinary and Animal Sciences, Lahore, Pakistan; 3 Department of Fisheries, Government of the Punjab, Lahore, Pakistan; 4 Aquaculture and Nutrition Research Unit (ANRU), Carna Research Station, Ryan Institute and School of Natural Sciences, College of Science and Engineering, University of Galway, Galway city, Ireland; University of Life Sciences in Lublin, POLAND

## Abstract

The bullseye snakehead (*Channa marulius*) is considered as an affordable and robust freshwater fish for farming in Asia. However, there is limited knowledge on the species’ full nutritional requirements to date with extensive gaps in our knowledge and particularly in precision aspects of protein requirements. Therefore, a three-month feeding trial was conducted under semi-intensive farming conditions to determine the protein requirement of bullseye snakehead using test diets containing 40 (P40), 45 (P45), 50 (P50), and 55% (P55) crude protein levels. The growth performance results revealed that the 55% dietary protein group (P55) had the highest final mean weight (14.09 g fish^-1^), and net weight gain (12.82 g fish^-1^). When compared to other dietary treatments, the final weight (R^2^ = 0.921), and weight gain (R^2^ = 0.913), displayed a linear increasing trend as dietary protein is raised. The lowest FCR was observed in 50% (1.94±0.01) and 55% (1.97±0.01) CP diet groups compared to dietary treatments. Further analysis has shown that the body protein content also significantly increased as dietary protein was raised to 55%. Although, a reverse trend was found in body lipid levels with increasing protein in the diet. The incremental dietary protein also elevated proximal intestinal protease activity but decreased amylase and lipase activity. The overall essential and non-essential amino acids levels of snakehead fillet muscle reflected an increase in dietary protein. Overall, this study has shown that the fish fed a diet with 55% crude protein attained the highest growth performance and nutrient profile of the whole fish when compared to other dietary treatments tested. It would appear we did not obtain the maximum potential for growth under the present experimental conditions due to the upper protein constraint of 55% in the diet. Further quantitative studies are suggested.

## Introduction

The giant snakehead (*Channa marulius*) is a commercially important freshwater fish commonly found in a variety of habitats, encompassing canals, rivers, swamps, marshes, ponds, reservoirs, lakes and rice fields in Pakistan, India, Thailand, Cambodia, and China [1]. *C*. *marulius* is locally known as ’Sole’ belonging to the family Channidae [2]. Due to its high meat quality and yield, longer shelf life, and has relatively high market demand. Furthermore, *C*. *marulius* is a viable species to fit in contemporary intensive rearing systems due to its improved adaptation to confinement, faster growth rates, survival and better feed conversion ratio [3]. Within Pakistan, three species of *Channa* are naturally found in the riverine systems, however these are not commercially farmed, i.e., *C*. *marulia*, *C*. *striata*, and *C*. *punctata*. More recently, the bullseye snakehead (*C*. *marulius*) has been introduced in Pakistan for farming. It is a highly attractive alternative to other snakehead species because of its larger size of up to 30 kg [4].

In order to fully realise the potential of bullseye snakehead for commercial production, it is important to understand their dietary requirements for optimum diet formulation. Artificial/compound feeds are the most effective to increase fish yields by providing all the necessary required nutrients for growth [5]. In particular, protein is the most important nutrient for fish growth, survival, and reproduction [6] forms the basis of the diet and is usually the most expensive component. Traditionally dietary protein is supplied through the use of fishmeal because of its similar biological value more importantly for carnivorous farmed fish species (inc. bullseye snakehead) due to its high protein value and palatability.

An earlier study in bullseye snakehead fry stage (~4 g to ~6 g) had shown that there was a positive dose-dependent relationship between dietary protein level and growth performance [7]. The results also revealed that the highest growth was attained through feeding the fish with a 60% (reported 58.7% after proximate analysis) protein diet. Protein requirements are generally higher for smaller fish. As fish grow larger, their protein requirements usually decrease. For other *Channa* species, the optimal protein requirement for the northern snakehead fish (*C*. *argus*) was 48% [8]. While another study found that the optimal dietary protein level was 55% in striped snakehead (*C*. *striata*) [9].

The gut enzyme profile is the indicator of nutrient digestibility and utilization. The composition of the diet (protein, carbohydrate and lipid) affects the activities of the digestive enzymes [10]. Along with exogenous enzymes (mainly produced by gut bacteria), endogenous enzymes play a vital role in digestion. A few researchers have been done on the study of digestive enzymes of freshwater fish species. Among several enzymes, proteases are the most crucial in carnivorous fish, as their diet contains protein-rich material which has a role in protein digestion. Carnivorous species, however, also exhibit endogenous amylase activity [11, 12]. While measured non-specific protease, amylase, and lipase activity in spotted sorubim catfish (*Pseudoplatystoma corruscans*) [13]. The effects of various dietary protein/carbohydrate ratios on the activities of enzymes involved in the metabolism of amino acids, as well as on Nile tilapia (*Oreochromis niloticus*) growth performance and body composition, were examined by Gaye-Siessegger et al. [14].

Due to the variation between species, stage of the life cycle, and the rearing environment, it is paramount to identify the optimal protein requirement with a level of specificity to avoid feed wastage and deterioration in the water quality. Therefore, the goal of the present study will be to assess the dietary protein requirement of *C*. *marulius* at the fry stage. The study will also examine the impact on the fish growth performance, digestive enzyme activity, body proximate composition, and the fillet muscle amino acid profile.

## Materials and methods

### Diet formulation and production

Four test diets were formulated to have protein concentrations of 40% (P40), 45% (P45), 50% (P50), and 50% (P55) using combinations of fish meal, maize gluten and casein as protein sources (Table 1). Wheat flour was used as a pellet binder and partial energy ingredient. Diet formulation and proximate composition are presented in Table 1. All the ingredients were chemically analyzed, weighed according to formulation and ground with a hammer mill, again weighed, and mixed in an electric food mixer (Kenwood KM 280). Water (15%) was added to create a dough mixturewhich was further pelletized by using a meat mincer ANEX AG3060. Pellets (1mm) were sun dried to about 10% moisture. The experimental feed was stored in at -20°C and used later in feed trial.

**Table 1:** Test diet formulation and proximate composition (%, dry weight).

*Diet formulation*	P40	P45	P50	P55
Fish meal^a^	30.00	30.00	30.00	30.00
Fish oil^a^	7.00	7.00	7.00	7.00
Casein^b^	10.00	15.00	20.00	25.00
Maize gluten ^c^	26.00	28.00	29.00	30.00
Wheat flour^d^	24.00	17.00	11.00	5.00
Vitamin premix^e^	1.00	1.00	1.00	1.00
Mineral premix^f^	1.00	1.00	1.00	1.00
Vitamin C^g^	1.00	1.00	1.00	1.00
***Proximate analysis***				
Moisture	9.23	8.86	8.82	9.02
Crude protein	40.00	44.90	49.58	54.95
Crude fat	8.75	8.82	8.92	8.89
Ash	12.07	11.56	9.46	8.90
Gross energy (MJ kg^-1^) ^h^	18.39	18.22	18.31	18.27
P/E ratio, (mg kJ^-1^)	21.40	22.56	24.83	27.19

a Paradise Fish meal, Karachi, Pakistan

b Casein (C17800-31), Unichem Pharmaceuticals Pakistan (Pvt) Ltd, Islamabad, Pakistan.

c Rafhan Custard Pvt. Ltd., Faisalabad, Pakistan.

d Family Flour Mill, Pattoki, Pakistan.

e Vitamin A 3,500,000 IU kg-1, vitamin B1 3,500 mg kg-1, Vitamin D3 1,750,000 IU kg-1, Zn gluconate 40 g kg-1, vitamin E 3.500 mg kg-1, vitamin PP (nicotinamide) 30g kg-1, sorbitol 20 g kg-1 (Fivevet, Central Veterinary Medicine JSC No. 5, Ha Noi, Vietnam).

f Ferrous sulphate 25 g kg-1, calcium phosphate 397 g kg-1, calcium lactate 327 g kg-1, magnesium sulphate 137 g kg-1, sodium chloride 60 g kg-1, potassium chloride 50 g kg-1, potassium iodide 150 mg kg-1, manganese oxide 800 mg kg-1, copper sulphate 780 mg kg-1, zinc oxide 1.5 g kg-1, cobalt carbonate 100 mg kg-1, manganese oxide 800 mg kg-1, sodium selenite 20 mg kg-1 (Fivevet, Central Veterinary Medicine JSC No. 5, Ha Noi, Vietnam).

g Vitamin C (ascorbic acid) 300 g kg-1 (Fivevet, Central Veterinary Medicine JSC No. 5, Ha Noi, Vietnam).

h Gross energy obtained through calorimetery.

### Ethical approval, fish, and feed trial

The protocols and procedures of this study were approved by the animal use and animal care committee of the University of Veterinary and Animal Sciences, Lahore, Pakistan (DR/163, 26-04-2021). Mixed-sex juvenile bullseye snakehead (*C*. *marulius*, ~1.0 g fish^-1^) were sourced from the Fish Biodiversity Hatchery (Chashma, District, Mianwali, Pakistan) and acclimated into recirculating circular tanks for 2 weeks at the Department of Fisheries and Aquaculture, Research and Training Facilities (UVAS, Ravi Campus, Pattoki, District Kasur, Pakistan). The fry was initially given live feed (Artemia and rotifers) for 5 days followed by a combination of live feed and egg shake for the next 9 days, then live feed was excluded from their diet. The feeding trial was carried out for 90 days in 12 HAPAS cages (150 (W) × 120 (L)× 90 cm (D); water depth 4 feet) which was installed in an earthen pond (half acre with 5 feet depth) filled by well water. According to requirements water in the pond was renewed and water exchange was undertaken every day in the pond as well as in the hapas. To prevent fish from jumping out and predatory birds, hapas were covered with nets throughout the experiment. Fish were fed on crumbled feed for the first 30 days. Then fry was fed with 1mm pellet size feed for the next 60 days. Each dietary treatment was fed to three replicate HAPA cages that were stocked with 15 fish (1.26 ±0.05 g). To assess the growth performance and adjusting the feed ration in response to increase weight gain, fish body weight was measured every fortnightly. The fish were fed three times a day (08:00, 13:00, 18:00) in equal portions at a feeding rate of 100% of the body weight. The feed ration level was adjusted down to 70% after day 30 and readjusted every 7 days to reach 40% of the body weight. During the feeding trial, the water temperature (33 ±0.27°C), dissolved oxygen (7.27 ±0.15 mg L^-1^), and pH (7.60 ±0.24) were monitored daily (Model 55, YSI Inc, Yellow Springs Ohio, USA).

### Sampling, growth performance and feed utilisation indices

The initial body weight (g) of *C*. *marulius* fry was recorded at the onset of the experiment and total of 15 fry in each of the twelve hapas were weighed every fortnightly to monitor growth and survival. The feeding experiment lasted for 90 days and at the end of the experiment, fish were starved for 24 hrs to allow gut clearance and fish from each replicate were harvested separately using fry harvesting net with a mesh size of 2mm. Fish were anaesthetized with clove oil (50 ml L^-1^) before sampling to minimize the possible stress induced during handling. Five fish were randomly collected from each experimental cage and were used for the analysis of whole-body proximate composition analysis while the intestine and muscles of the other five fish from each experimental cage were dissected for the analysis on digestive enzyme activities and muscles amino acid profile. To assess the effects of increasing dietary protein on snakehead growth performance, the following calculations were used:

$$Mean\;weight\;gain\left(\text{g}\right)=\text{final}\;\text{body}\;\text{weight}\;\left(\text{g}\right)-\text{initial}\;\text{body}\;\text{weight}\;\left(\text{g}\right)$$ (1)

$$Specific\;growth\;rate\;SGR\left(\%\;\text{day}-1\right)=\left[\left(\text{Final}\;\text{weight}-\text{Initial}\;\text{weight}\right)/\text{days}\;\text{of}\;\text{growth}\;\text{trial}\right]\times 100$$ (2)

$$Feed\;conversion\;ratio\left(\text{FCR}\right)=\text{Feed}\;\text{intake}\;\left(\text{g}\right)/\text{weight}\;\text{gain}\;\left(\text{g}\right)$$ (3)

### Proximate composition

The proximate analysis of test diets and fish body were carried out from each replicated fish group. Samples were homogenised using a domestic food blender. The moisture, protein, lipid, and ash contents were analyzed following the protocols of the Association of Official Analytical Chemists [15]. Moisture was determined by drying at 105°C until a constant weight was achieved. Similarly, ash was heated to 550°C for 16 hours to a constant weight furnace (Tmf-3100, Eyela Co, Tokyo, Japan). Protein content was measured using the Kjeldahl procedure (Kjeltec 8100, FOSS, Hilleroed, Denmark). Crude fat was assessed by Soxhlet extraction (R106S, Behr Labor-Technik, Düsseldorf, Germany) using petroleum ether.

### Digestive enzyme activity

After the feeding trial, five fish from each replicate were randomly sampled and the intestine was dissected and homogenised in Tris HCl buffer (0.1 M, pH 7.4, 1:9 ratio) for 15 minutes at 4°C on a tissue lyser at 6,000 *g*. The lipid fraction was removed, and the supernatant was recovered and stored at -20°C for later enzyme activity determination. The activities of amylase, protease, and lipase were carried out as described by [16]. Intestinal protease enzyme activity was determined by making a substrate solution of 1% azoalbumin in 50 mM Tris-HCL (pH 7.5). The enzyme extract of 10 μL was taken and mixed with buffer (0.5 mL; pH 7.5) and then 0.5 mL substrate solution was added. The samples were incubated at 25°C for 10 min. The reaction was stopped by adding trichloroacetic acid 0.5 mL and centrifuged at 14,000 *g* for 3–5 min. The absorbance of collected supernatant aliquots was read at 366 nm [17]. To analyse the amylase activity, 1 mL of supernatant was mixed with 2 mL of starch phosphate buffer and incubated at 37°C for 30 min. After that 3 mL of DNS solution was added and again incubate until a brown color appeared. The solution was diluted by adding 4 mL of water to make 10 mL of volume. The optical density of the supernatant was measured at 540 nm [18]. For Lipase activity, 50 μL of the extract solution was mixed with 0.5 mL olive oil, 10 mM CaCl_2_ and phosphate buffer 0.5 mL. Samples were incubated at 30˚C for 30 min and the reaction was stopped by adding 20 mL of ethanol. The fatty acids were estimated by 50 mM KOH [19]. Pepsin activity was assayed by using haemoglobin as the substrate at 280 nm [20]. Trypsin activity was measured by using the substrate benzoyl-Arg-p-nitroanilide (BAPNA) [21]. BAPNA (1.0 mM) was prepared by dissolving it in 1.0 mL DMSO, and then adding 50 mM TRIS buffer (pH-7.5) containing 20 mM CaCl2 to make 100 mL. The experiment was carried out at a temperature of 37°C. After incubating for 10 minutes with 10 mL enzyme preparation 1.25 mL substrate solution, 0.25 mL acetic acid (30%) was added. As a control, absorbance was measured at 410 nm against a water blank. Chymotrypsin activity was measured by using substrate succinyl-(Ala)2-Pro-Phe -p-nitroanilide (SAPNA) [22]. The substrate solution (100 mL of 0.02 mM) was mixed with 590 mL of 0.1 M TRIS (Ph-7.8), with 0.01 M CaCl_2_ and was mixed with 10 mL of the enzyme solution, and the absorbance was measured after 3 minutes at 410 nm.

### Amino acid composition

The amino acid profile of the test diets and fish muscle fillet (*n* = 3) were analysed by ion-exchange chromatography. Samples (~3 g) were homogenised and graded <500 μm. To protect the lysine and methionine from oxidation, samples were treated with 5 mL formic acid to lysine-lysine acid and met-methionine sulphone. Samples were hydrolysed with 25 mL 6 M HCl/phenol for 24 hours at 110°C and samples were subsequently adjusted to pH 2.2. The sample solution was filtered, and the amino acids were measured on a Biochrom 30+ amino acid analyser (Cambridge, United Kingdom). The amino acid profile on the basal diets is shown in Table 2 and summated as both essential and non-essential amino acids amounting to the total crude protein of the respective diets. The elevated total amino acids reflected the increasing level of dietary protein but did now vary as a percentage of the protein (% of crude protein).

**Table 2:** Amino acid profile of the bullseye snakehead test diets (%, dry matter).

Amino acids	P40	P45	P50	P55
***Essential amino acids*, *EAA % of diet DM***				
Arginine	1.55	1.98	2.32	2.48
Histidine	1.07	1.12	1.16	1.22
Isoleucine	2.12	2.49	2.57	2.81
Leucine	2.93	3.24	3.66	3.87
Lysine	3.22	3.75	4.09	4.23
Methionine	1.02	1.08	1.11	1.14
Phenylalanine	2.01	2.04	2.12	2.16
Threonine	2.08	2.13	2.22	2.41
Valine	2.02	2.05	2.32	2.16
***Sum of EAAS***	18.02	19.88	21.57	22.48
***Non-essential amino acids*, *NEAA***				
Alanine	1.48	1.89	2.03	2.15
Aspartic acid + Asparagine	4.13	4.23	5.01	5.12
Cysteine	2.11	2.41	2.53	2.74
Glutamic acid + Glutamine	4.03	4.17	4.19	4.24
Glycine	3.06	3.28	4.04	4.42
Proline	1.24	1.49	2.01	2.23
Serine	2.02	2.07	3.15	3.32
Tyrosine	1.01	1.19	2.24	2.29
Ornithine	0.87	1.06	1.18	1.28
***Sum of NEAAS***	19.95	21.79	26.38	27.79

### Data analysis

Datasets are presented as means with the corresponding standard deviation. Datasets were analysed using one-way analysis of variance (ANOVA). To determine significant differences between treatment diets, *post hoc* Duncan’s Multiple Range Test was carried out. Permutational ANOVA (PERMANOVA) was performed using Euclidean Distances to assess the dietary protein treatment effect on fish amino acid profile (randomisation based on 9999 permutations). Furthermore, principal component analysis was carried out to visualise and understand the amino acid changes. A statistically significant difference in a tested parameter was only considered when p <0.05.

## Results

### Growth parameters

After the 90-day feeding trial, fish had grown at least over eight-fold in weight gain. The increasing dietary protein inclusion had a general impact on the growth performance snakehead fish (Table 3). The highest final mean weight and weight gain were found in fish that were fed with the 55% protein diet (P55) when compared to any other treatment diet (p<0.05). This effect was also reflected in the SGR values with P40 having the lowest value and P55 being the highest SGR value, (p<0.001). Overall, the results showed that the treatment diets produced a linear dose-response relationship in the final mean weight (y = 0.257x + 0.053, R^2^ = 0.921), and weight gain (y = 0.256x + 1.130, R^2^ = 0.913). Since the growth response did not plateau, no break-point could be established or application of a quadratic orpolynomial equation to estimate the protein requirement in this case. The attainment of lower FCR was observed in the CP50 diet group, but both CP55 and CP45 still had significantly lowered FCR values compared the CP40 diet (P<0.001) indicative of superior feed conversion efficiency at higher protein intake.

**Table 3:** Growth performance and feed utilisation indices of bullseye snakehead (*Channa marulius*) fish fed with increasing dietary protein levels for 90 days (n = 3 replications).

Parameters	P40	P45	P50	P55	P-value
Initial mean weight; g fish^-1^	1.23±0.05	1.27±0.07	1.26±0.06	1.26±0.06	0.872
Final mean weight; g fish^-1^	10.57±0.12^d^	11.08±0.07^c^	13.39±0.39^b^	14.09±0.09^a^	<0.001***
Mean weight gain; g fish^-1^	9.33±0.08^a^	9.81±0.13^b^	12.13±0.44^c^	12.82±0.15^d^	<0.001***
FCR; g g^-1^	2.41±0.00^d^	2.16±0.00^c^	1.94±0.01^a^	1.97±0.01^b^	<0.001***
SGR; %	2.38±0.03^a^	2.40±0.06^a^	2.62±0.08^b^	2.67±0.05^b^	<0.001***
Survival rate; %	81.33±6.02^a^	92.93±2.60^b^	84.66±5.50^a^	93.36±1.73^b^	0.022*

± SD. Different superscripts on the same row indicates there is a significant difference (P<0.05).

* P<0.05,

** P<0.01,

*** P<0.001. Diets: 40% (P40), 45% (P45), 50% (P50), 55% (P55) dietary protein level.

### Proximate body composition

Significant differences were observed between dietary treatments for all measured proximate composition parameters in the harvested fish: moisture, protein, lipid, and ash (p<0.05) are shown in Table 4. Moisture content had significantly decreased at both 45 and 50% protein inclusion level (p<0.001). The crude protein content in fish was significantly improved by dietary treatment levels (p<0.05). The highest values for crude protein (20.06%) were recorded in fish fed P55 (55%) diet than other dietary treatments. However, the higher protein level in the diet (55%, P55) did not show a significant difference to the 50%, (P50) treatment group. While body lipid content showed a general inverse trend as dietary protein increased (3.08 >1.93%). Body ash concentration was at its highest in fish fed with 45% (P45) protein level but declines back to a lower level when dietary protein increased which is similar to the 40% protein diet group (P45, p<0.001).

**Table 4:** The body composition of bullseye snakehead (*Channa marulius*) fish after being fed with increasing protein levels diets for 90 days (%, wet basis, n = 3 replications).

**Parameters**	**P40**	**P45**	**P50**	**P55**	**P-value**
Moisture	76.31±0.60^b^	75.81±0.21^b^	75.92±0.17^b^	74.38±0.14^a^	0.001**
Protein	17.08±0.43^a^	17.85±0.44^a^	19.88±0.66^b^	20.06±0.68^b^	<0.001***
Lipid	3.08±0.07^b^	2.91±0.05^b^	1.84±0.11^a^	1.93±0.06^a^	0.007**
Ash	4.35±0.38^ab^	4.92±0.06^b^	3.72±0.15^a^	3.81±0.12^a^	0.022*

± SD. Different superscripts on the same row indicate there is a significant difference.

* P<0.05,

** P<0.01,

*** P<0.001. Diets: 40% (P40), 45% (P45), 50% (P50), and 55% (P55) dietary protein level.

### Amino acid profile

**Fig 1:**
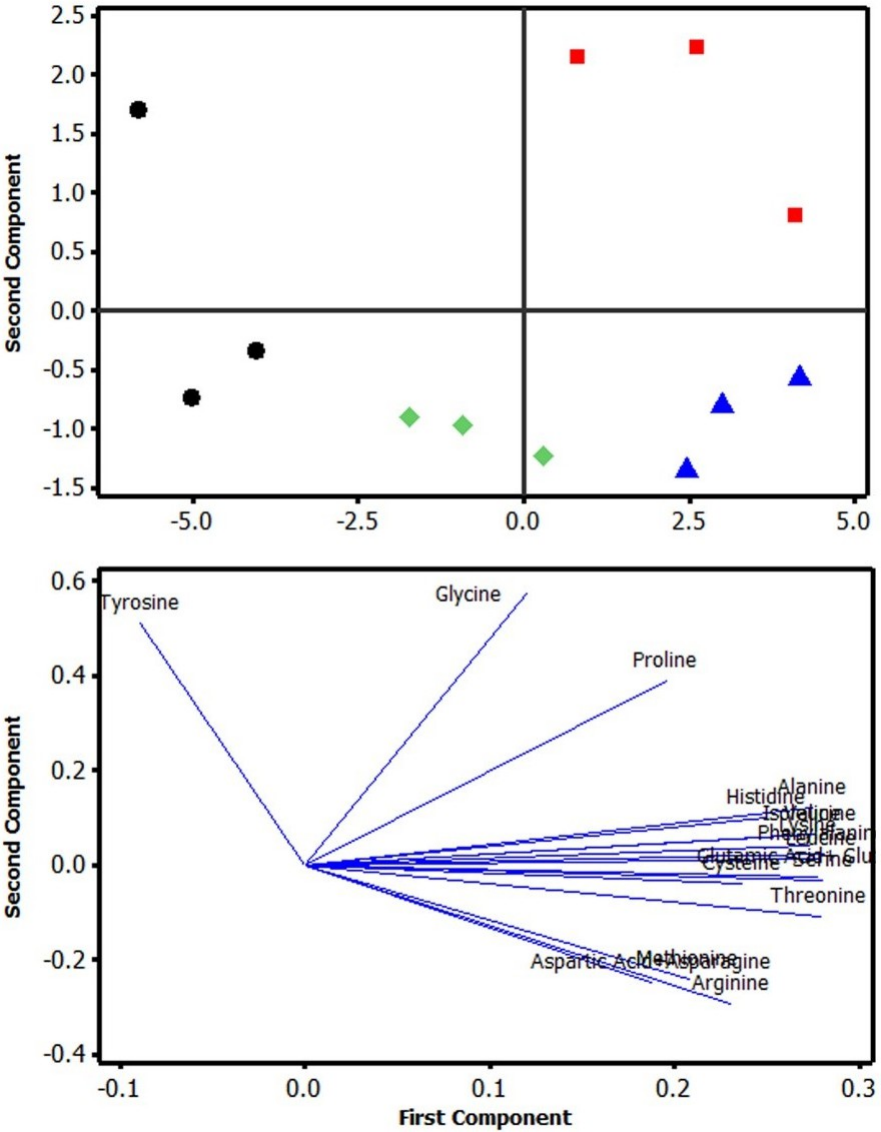
Principal component analysis of the muscle amino acid composition. Score biplot (A) and loading plot (B) of the first and second principal components for amino acid between amino acid and dietary treatment. The first and second principal component explains 83.50% of the sample variation. Diets: 40% (●), 45% (■), 50% (♦), 55% (▲) dietary protein level.

The results for the amino acid profile of bullseye snakehead fed on different levels of protein are given in Table 5. In the current study, a total of eighteen amino acids were analyzed from which sum of essential and non-essential amino acid concentration in the muscle of fish fed P55 (55%) protein was significantly higher than that of fish fed with P40, P45, or P50 protein diets. Dietary protein levels significantly affected the content of lysine, methionine, and valine (p<0.05), while no significant changes were observed in other essential amino acids content (p>0.05). Furthermore, no significant differences was found in serine measured between the treatments (p>0.05), while alanine, aspartic acid, cysteine, glutamic acid, glycine, proline, tyrosine, and ornithine content in non-essential amino acids were significant (p<0.05). For glutamic acid + glutamine, there was an increasing trend in measured levels as dietary protein had increased, which led to P55 having the highest content in comparison to other treatment groups (p<0.05). The difference in fish muscle amino acid composition is also shown in the principal component analysis, with each dietary protein level are spatially separated into discrete groups in the score biplot (Fig 1A). Furthermore, the loading of the plot of the results revealed that tyrosine, glycine, and proline seem to independently increase when compared to other amino acids (Fig 1B).

**Table 5:** Amino acid profile of bullseye snakehead (*Channa marulius*) fish muscle after being fed with increasing levels of protein diets for 90 days (%, dry matter, n = 3 replications).

Amino acids	P40	P45	P50	P55	P-value
***Essential amino acids*, *EAA (%DM)***					
Arginine	4.68±0.41^a^	4.85±0.09^a^	4.73±0.79^a^	4.76±0.31^a^	0.977
Histidine	1.11±0.11^a^	1.55±0.38^a^	1.16±0.10^a^	1.25±0.19^a^	0.175
Isoleucine	2.68±0.40^a^	2.65±0.29^a^	2.41±0.23^a^	2.69±0.19^a^	0.628
Leucine	4.41±0.28^a^	4.69±0.13^a^	4.67±0.35^a^	4.72±0.15^a^	0.437
Lysine	3.70±0.32^a^	3.57±0.30^a^	4.88±0.27^b^	5.06±0.12^b^	<0.001***
Methionine	2.98±0.23^a^	3.93±0.20^bc^	3.66±0.30^b^	4.37±0.24^c^	0.001**
Phenylalanine	2.87±0.15^a^	3.25±0.37^a^	2.67±0.39^a^	2.87±0.17^a^	0.192
Threonine	1.49±0.27^a^	1.71±0.28^a^	1.51±0.16^a^	1.57±0.26^a^	0.720
Valine	2.71±0.30^a^	2.92±0.14^a^	2.92±0.10^a^	3.05±0.30^a^	0.397
***Sum of EAAS***	26.66±0.84^a^	29.14±0.24^b^	28.63±1.39^b^	30.37±1.10^b^	0.011*
***Non-essential amino acids*, *NEAA***					
Alanine	4.05±0.17^a^	4.72±0.17^b^	4.65±0.22^b^	4.19±0.08^a^	0.003**
Aspartic acid + Asparagine	3.39±0.21^a^	3.42±0.08^a^	4.26±0.03^b^	4.53±0.32^b^	<0.001***
Cysteine	0.39±0.08^a^	0.64±0.08^b^	0.53±0.07^ab^	0.54±0.08^ab^	0.037*
Glutamic acid + Glutamine	6.87±0.29^a^	7.08±0.14^a^	8.79±0.22^b^	9.43±0.24^c^	<0.001***
Glycine	5.67±0.34^ab^	5.81±0.29^b^	5.33±0.21^ab^	5.21±0.19^a^	0.074
Proline	4.34±0.35^ab^	4.40±0.33^b^	3.81±0.25^a^	3.93±0.14^ab^	0.086
Serine	3.28±0.35^a^	3.18±0.17^a^	2.91±0.10^a^	3.02±0.07^a^	0.201
Tyrosine	0.30±0.06^c^	0.34±0.07^c^	0.21±0.02^b^	0.09±0.01^a^	0.001**
Ornithine	2.12±0.30^a^	2.23±0.22^a^	2.82±0.22^b^	3.01±0.13^b^	0.003**
***Sum of NEAAS***	30.44±0.43^a^	31.85±0.21^b^	33.34±0.59^c^	33.99±0.48^c^	<0.001***

Different superscripts on the same row indicates there is a significant difference. ± SD.

* P<0.05,

** P<0.01,

*** P<0.001. Diets: 40% (P40), 45% (P45), 50% (P50), 55% (P55) dietary protein level.

### Digestive enzymes activity

**Fig 2:**
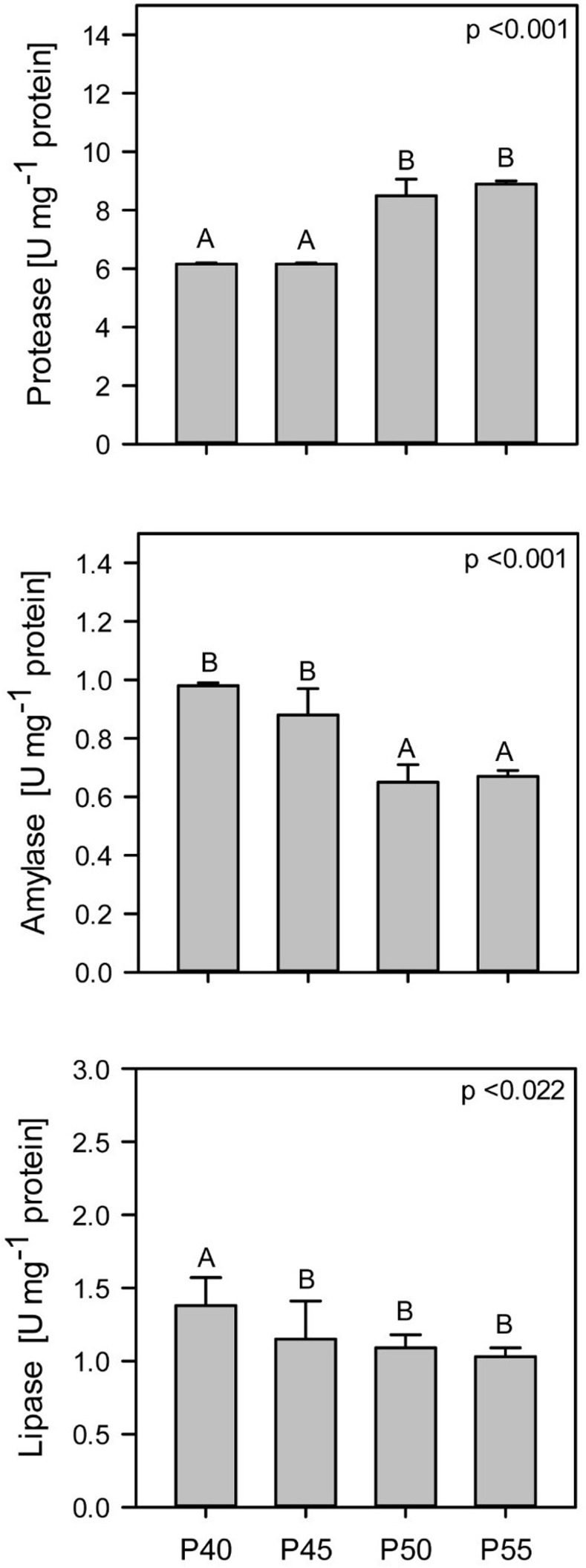
Intestinal enzyme activity in snakehead (*Channa marulius*) fish fed with increasing levels of protein (± SE). Diets: 40% (P40), 45% (P45), 50% (P50), 55% (P55) dietary protein level. Different superscripts indicate there is a significant difference (P<0.05).

**Fig 3:**
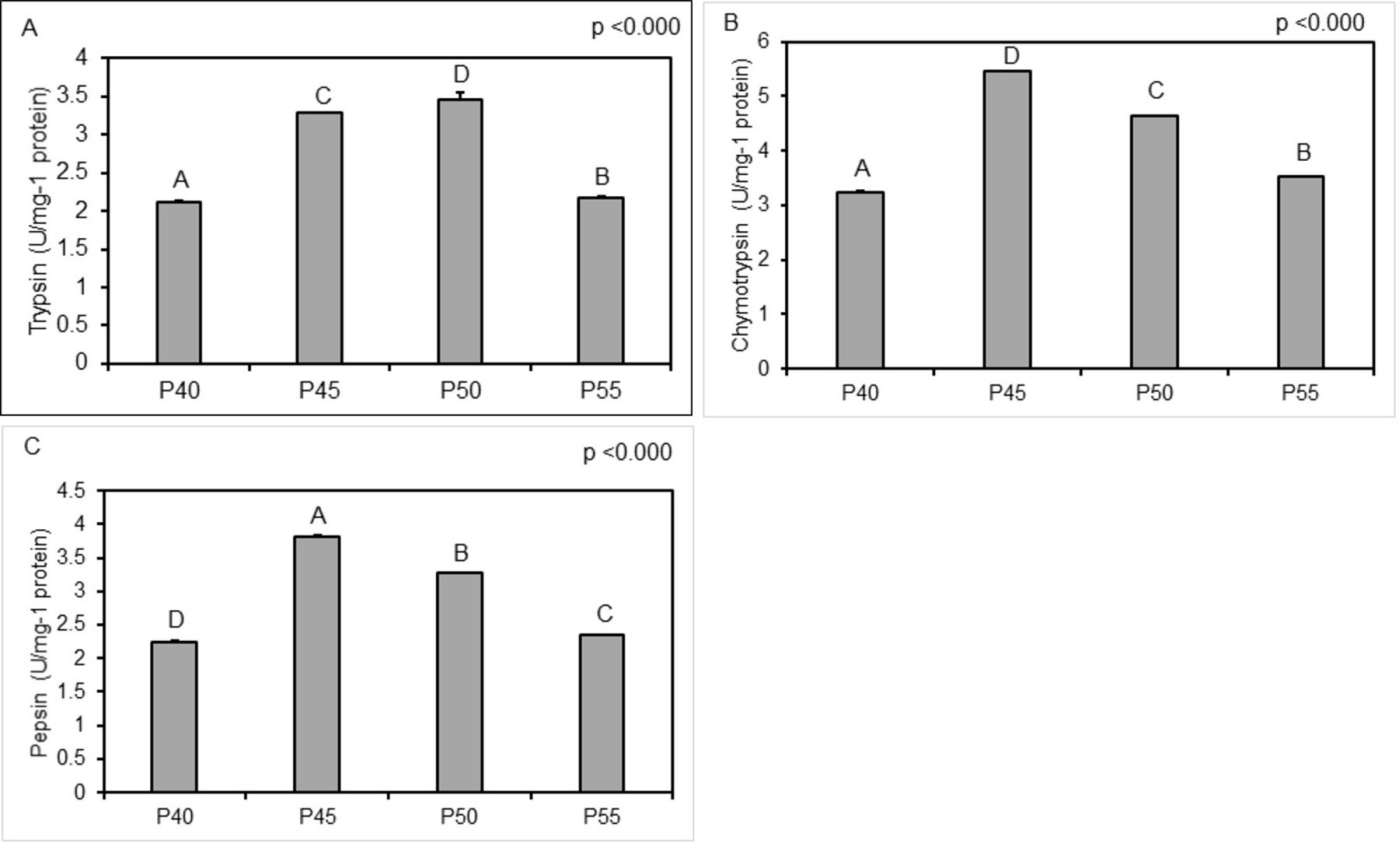
**(A-C)**. Intestinal enzyme activity in snakehead (*Channa marulius*) fish fed with increasing levels of protein (± SD). Diets: 40% (P40), 45% (P45), 50% (P50), 55% (P55) dietary protein level. Different superscripts indicate there is a significant difference (P<0.05). A, B & C; trypsin, chymotrypsin, and pepsin, respectively.

The intestinal digestive enzyme activities of bullseye snakehead were significantly affected by dietary protein (casein) levels (Fig 2). Specifically, protease activity had significantly increased by >37% at 50% (P50), and 55% (P55) dietary protein levels when compared to P40 (40%) and P45 (45%) treatment groups (p<0.001). In contrast, both amylase and lipase enzyme activities showed a general decreasing trend as dietary protein levels increased. However, the decrease is only significantly different when dietary protein level was increased to and beyond 50% (P50, p<0.001) for amylase and 45% (P45, p<0.022) for lipase activity. The activities of trypsin, chymotrypsin and pepsin are presented in Fig 3. Significantly highest trypsin activities were measured in P50, followed by P45, P40, and P55 groups (p<0.05). Furthermore, both chymotrypsin and pepsin activities recorded the highest values in both P45 and P50 groups but decreased significantly in P55 and P40 groups, respectively.

## Discussion

The bullseye snakehead is a carnivorous fish, that naturally preys upon other fish, amphibians, and insects [8]. The growth rate of bullseye snakehead is comparable to other *Channa* species and is usually linked positively to the dietary protein levels since protein is a driver for carnivorous fish for both tissue accretion (e.g., muscle building) and as an energy source [8, 23]. It is essential to meet the dietary protein requirement in farmed fish to optimally grow, but not in excess where the protein is not put into growth and leading to unnecessary feed costs.

The present feeding trial utilised casein and fish meal as the protein sources in the formulated diets and demonstrated that 55% dietary protein resulted in the highest weight gain for bullseye snakehead juveniles. Growth was not significantly altered in fish fed different protein inclusions (40–55%). No significant differences or patterns in SGR were observed between dietary treatments. Raizada et al. [7] also observed no significant difference in SGR between treatments. This contrasts with Mohanty et al. [9] that found significant growth between diets of differing protein levels up to 550 g kg^-1^ (i.e., 55%) inclusion, after which SGR began to decrease. Kpogue et al. [24] reported similar results, with final body weight and SGR increasing from 35 to 55% with increased dietary protein for obscure snakehead (*Parachanna obscura*) larvae. The results of the present study indicate a linear response to growth within the narrow protein range tested for juvenile snakehead. It is clear that maximum response was not attained and would have been observed above 55% dietary crude protein level. An optimum protein requirement was therefore not feasible within the constraints of this study. A further experiment would be necessary with a protein range from 55% to >65% to confirm this for juvenile snakehead. However, since there is a requirement for protein to meet both growth (protein retention) and energy the study should also ideally test protein to energy ratios in such a study.

Protein provides a proportionally significant component of fish tissues and organs, especially during the early growth phase. A similar study on bullseye snakehead supports that protein requirement is higher at the fry stage [7]. Inadequate levels of protein in the diet will inevitably lead to reduced growth and weight loss. A 2017 study observed similar findings and recorded the highest growth with a 52.5% protein diet and the lowest with a 38.8% protein diet on juvenile catfish [25]. The optimum protein level for snakehead (*C*. *striatus*) fry to fingerling stage based on weight gain and survival was estimated to be 45% reported by Yeshdas et al. [26]. Furthermore, the optimum protein level for young stinging catfish (*Heteropneustes fossilis*) based on growth performance was estimated to be 40–43% reported by Siddiqui et al. [27]. The difference in protein requirements among these species could be related to the different dietary formulations, environmental conditions, fish species, age class, and different methodologies applied [28–30].

In this study, whole-body protein content increased as dietary protein was raised. However, there is an indication that the rate of increase in body protein stabilises above the 50% dietary protein treatment group. This finding suggests that protein retention efficiency is close to attaining its maximum physiological capacity for this species. Numerically higher values of crude protein were reported in fish fed with the 55% protein diet, which played a major role in improving fish growth compared to lower protein diets, i.e., 40, 45, and 50%. This suggests that net protein utilisation efficiency was higher in this treatment group. Although this was not possible to confirm as no initial fish composition was analysed. These findings are consistent with those of Raizada et al. [7] that found protein efficiency was directly proportional to the protein content across all levels of treatment. Another study on striped snakehead (*C*. *striatus*) fingerlings has reported similar outcomes Aliyu‐Paiko et al. [31], as well as on dietary protein requirement of juvenile cachara catfish (*Pseudoplatystoma reticulatum*) [32].

An inverse relationship of lipids is found in the body composition, where lipid content decreased as dietary protein increased, even though all diets are iso-lipidic and used the same proportion of oil to make the diet (i.e., fish oil). In comparison to Raizada et al. [7] study on the fry stage of bullseye snakehead, there was no general trend of decreasing body lipid content. Instead, there was a rise of lipids in the higher dietary protein treatments, i.e., 53% and 59% protein treatments. While the spotted snakehead (*C*. *punctatus*) had increasing body lipid content when dietary protein was increased up to 55% [33]. Our investigation may have recorded more lipid energy being partitioned for protein accretion in growth and also to provide energy for de-amination and nitrogen excretion with increasing dietary protein levels thus progressively reducing total fat storage in snakehead.

The present study also found that both moisture and ash levels were different between the treatment diets. However, there was no apparent trend in these changes relating to the dietary protein content being given. When compared to other *Channa* protein requirement studies, there the results for moisture and ash were also variable. For instance, Raizada et al. [7] found that there were variable differences in the moisture content of bullseye snakehead but no trend indicating a dose-response effect from the increase in dietary protein. While Zehra and Khan [33] showed a rising moisture content in spotted snakehead with increasing protein levels in the diet, but no differences were observed between the different treatment body ash levels.

The highest concentration of essential amino acids was found for lysine (5.06%). Such findings are comparable to Pratama et al. [34], who also stated the most important amino acid for the giant snakehead (*Channa micropeltes*) was lysine (2.02%, live wet weight). Various studies have confirmed the role of lysine on growth and development in fish [35]. In addition, lysine is one of the major essential amino acids,- which can induce albumin synthesis [36]. In this study, the highest non-essential amino acid was glutamate (9.43%) which was comparable to those reported by Zuraini et al. [37]. Glutamate is a key source of nitrogen in the *de nov*o synthesis of the non-essential amino acids for the rapid turnover of the enterocyte absorptive intestinal epithelium in fish as well as in other higher animals [38]. Although not deemed to be significant, there was an interesting rise in the level of ornithine for bullseye snakehead peaking at 55% protein relative to the lower protein diet-fed group (2.12 to 3.01%). Furthermore, urea is not the major product of ammonia detoxification in *C*. *asiatica*, which does not possess a functioning ornithine urea cycle [39]. Snakehead fish (*Channa* sp.) is thus considered to be ammoniotelic fish with a deamination route for excess nitrogen excretion principally as ammonia or ammonium into the water column.

In this study, the fish muscle amino acid profile was altered by protein intake. Absorbed amino acids may be selectively channelled into other metabolic demands at the higher intake including being oxidized for energy in ketogenic pathways and some contributing to gluconeogenesis [40]. It is likely that the muscle profile for EAAs and NEAAs would remain stable compared to the overall amino acid systemic pool. In this study, the total amino acid profile was measured but not for selected organs, including the liver. This has not been properly established for the snakehead genus (*Channa* spp.). Differential dietary protein levels and resulting protein accretion in muscle can provide information on the qualitative and quantitative EAA composition.

The intestinal digestive enzyme activity was influenced by the dietary regimes of fish sampled at termination of the feed trial. The present study addressed the distribution of protease, amylase, lipase, trypsin, chymotrypsin and pepsin in the intestinal proximal part of *Channa marulius*. It is widely recognised that the digestion of protein is a complex process in fish and occurs not only in the stomach compartment but also in other areas of the digestive system such, as the pyloric caeca and mid-intestinal region [41]. The higher growth efficiency and elevated protease activity in the current study were observed in the treatment group (P55). High acidic protease activity in the stomach of bagrid catfish is considered an indication of pepsin activity, as has been reported in other carnivorous fish [42, 43]. The occurrence of trypsin and chymotrypsin was detected in carnivorous species [44, 45]. As a carnivorous fish species, the bullseye snakehead is adapted to a high dietary protein intake, and as such shows a comparable lipase and lower amylase activity relative to other species. Amylase is a necessary enzyme for starch hydrolysis and responds to dietary carbohydrate levels. In other carnivorous fish such as salmon and trout, intestinal amylase activity may show some adaptation to a moderate dietary inclusion of carbohydrates with respect to starch digestion over time [46]. It would be interesting to examine these enzymes in further work on bullseye snakehead in response to experimental diets with varying protein levels and different protein-to-non-protein (i.e., lipid and starch) energy ratios. According to earlier studies, the dietary supplementation of enzymes capable of hydrolyzing starch and protein can further promote the body’s digestion and absorption of nutrients once they reach the digestive tract, by acting as a nutrient substrate for some endogenous enzymes, and promoting the secretion of endogenous enzymes [47, 48]. Contrarily, adding exogenous enzyme to the diet of sea bass did not increase the activities of digestive enzymes [49], and adding the NSP enzyme complex to the diet of the hybrid tilapia diet had no impact on the activities of protease and lipases [50]. This disparity between the studies mentioned above could be explained by variations in exogenous dietary supplementation and fish species.

It should also be noted that a series of semi-purified experimental type diets were used in the present investigation to achieve homogeneity of ingredients and to minimise the potential interactive effects of other feed ingredients. Fishmeal was the primary protein source together with casein. In practical diets, it will be necessary to examine feeds formulated from local raw materials and to assess their amino acid profile and the individual specific digestibility coefficients. More importantly, the overall formulated feed and protein digestibility. These factors will certainly influence the optimum crude protein requirements for the species if diets are formulated on a gross protein consideration, and not based on apparent digestible values for protein. Furthermore, preferably individual amino acids as employed by the poultry industry for finely balanced diets. The cost of the overall feed is another factor to be considered. Obtaining the desired optimum dietary protein level in diets will dictate the economics of intensive juvenile production and grow-out stages for bullseye snakehead in Pakistan and other snakehead farming regions.

## Conclusion

Considering current experiment results, dietary protein levels significantly improved growth performance, feed conversion ratio, assimilation of dietary amino acid, and body composition of juvenile bullseye snakehead. Furthermore, it was observed that specific enzyme digestive activities in juvenile bullseye snakehead can be modulated by the level of dietary protein. The results suggest that the ideal dietary protein requirement was at 55% in a semi-purified diet formulation when using growth and feed utilization parameters as the performance indicator. However, the growth performance indices could potentially be higher if the dietary protein were raised beyond 55%. Overall, this study has shown that the fish fed a diet with 55% crude protein attained the highest growth performance and nutrient profile of the whole fish when compared to other dietary treatments tested.
